# Mucoepidermoid Carcinoma of the Salivary Gland: Demographics and Comparative Analysis in U.S. Children and Adults with Future Perspective of Management

**DOI:** 10.3390/cancers15010250

**Published:** 2022-12-30

**Authors:** Asad Ullah, Jaffar Khan, Abdul Waheed, Nabin Raj Karki, Mya Goodbee, Abdul Qahar Khan Yasinzai, Bisma Tareen, Agha Wali, Khaleel Ahmad Khan, Muhammad Samsoor Zarak, Israr Khan, Andrea Agualimpia Garcia, Adil Khan, Marjan Khan, Sana Jogezai, Junaid Ahmad, Luis Velasquez Zarate, Nikhil Patel, Nagla Abdel Karim, Saleh Heneidi

**Affiliations:** 1 Department of Pathology and Laboratory Medicine, Vanderbilt University, Nashville, TN 37232, USA; 2 Department of Pathology, Indiana University School of Medicine, Indianapolis, IN 46202, USA; 3 Department of Surgery, San Joaquin General Hospital, French Camp, CA 95231, USA; 4 Department of Hematology and Medical Oncology, University of South Alabama, Mobile, AL 36688, USA; 5 Medical College of Georgia, Augusta University, Augusta, GA 30912, USA; 6 Department of Medicine, Bolan Medical College, Quetta 83700, Pakistan; 7 Internal Medicine, Howard University Hospital, Washington, DC 20060, USA; 8 Hackensack Meridian Health, Palisades Medical Center, North Bergen, NJ 07047, USA; 9 Department of Pathology & Immunology, Baylor College of Medicine, Houston, TX 77030, USA; 10 Division of Infectious Diseases, University of Louisville, Louisville, KY 40202, USA; 11 Marshfield Clinic, Marshfield, WI 54449, USA; 12 Department of Pathology, Medical College of Georgia, Augusta University, Augusta, GA 30912, USA; 13 Inova Schar Cancer Institute, University of Virginia, Fairfax, VA 22031, USA; 14 Department of Pathology, Cedars Sinai Medical Center, Los Angeles, CA 90048, USA

**Keywords:** salivary gland neoplasms, mucoepidermoid carcinoma, SEER database, poorly differentiated

## Abstract

**Simple Summary:**

Salivary gland tumors are rare among both pediatric and adult populations. Mucoepidermoid carcinoma (MEC) is a common type of malignant salivary gland tumor that presents with atypical clinical features. It is more commonly found in adults. Most commonly, MEC was less than 2 cm in size, moderately differentiated, and localized to the gland. Surgical resection was the most common treatment modality in both pediatric and adult populations (53.5%). The pediatric population with MEC had a lower death rate compared to the adult population. Tumor size greater than 2 cm, male sex, and distant spread were factors associated with a higher risk of death.

**Abstract:**

Background: Salivary gland neoplasms are uncommon in both pediatric and adult populations. Mucoepidermoid carcinoma (MEC) is one of the most common salivary gland tumors and usually presents with atypical clinical features. This study sought to evaluate the demographic and clinical factors affecting outcomes in adults and pediatric populations with MEC that could be used to risk stratification for treatment selection and clinical trial enrollment. Methods: Data on 4507 MEC patients were extracted from the Surveillance Epidemiology and End Result (SEER) database (2000–2018). Patients aged ≤ 18 years were classified into the pediatric population, and those older than 18 years were placed in the adult group. Kaplan–Meier survival curves were created to analyze survival probabilities for various independent factors. Results: The pediatric population comprised 3.7% of the entire cohort, with a predominance of females (51.5%), while the adult population constituted 96.3% of the cohort, with a predominance of female patients (52.2%). Caucasians were the predominant race overall (75.3%), while more African Americans were seen in the pediatric group. In tumor size of <2 cm overall, poorly differentiated tumors with higher metastasis rates were observed more in adults (11.3% and 9.3%) than in the pediatric population (3.0% and 4.8%, *p* < 0.05). Surgical resection was the most common treatment option (53.9%), making up 63.6% of the pediatric and 53.5% of the adult groups. A combination of surgical resection and radiation was used in 29.8% of the entire cohort while a combination of surgical resection, radiation, and chemotherapy made up only 3.2%. The pediatric group had a lower overall mortality rate (5.5%) than the adult group (28.6%). Females had a higher 5-year survival rate in comparison to males (86.5%, and 73.7%, respectively). Surgical resection led to a more prolonged overall survival and 5-year cancer-specific survival (98.4% (C.I, 93.7–99.6) in the pediatric group and 88.8% (C.I, 87.5–90.0) in the adult group), respectively. Metastasis to the lung, bone, brain, and/or liver was found to have significantly lower survival rates. Multivariate analysis demonstrated that adults (hazard ratio [HR] = 7.4), Asian or Pacific Islander (HR = 0.5), male (HR = 0.8), poorly differentiated histology (HR = 3.8), undifferentiated histology (HR = 4.5), regional spread (HR = 2.1), and distant spread (HR = 3.2) were associated with increased mortality (*p* < 0.05). Conclusions: Mucoepidermoid carcinoma of the salivary glands primarily affects Whites and is more aggressive in adults than in the pediatric population. Even with surgical resection, the overall survival is poor in the adult population as compared to its pediatric counterparts. Advanced age, larger tumor size, male sex, and lymph node invasion are associated with increased mortality.

## 1. Background

Salivary gland neoplasms are rare in both pediatric and adult populations and demonstrate considerable histological, biological, and clinical diversity [1]. In the general population, 1% of all cancers are salivary gland neoplasms, with 5% occurring among children < 20 years of age [2]. In the United States (US), salivary gland neoplasms account for 11% of all oropharyngeal neoplasms [3]. In children, salivary gland neoplasms comprise 8–10% of the pediatric population with head and neck malignancies [4].

The parotid gland is the most common site for benign and malignant salivary gland tumors, followed by the minor salivary glands of the oral cavity, submandibular glands, and rarely, sublingual glands. Mucoepidermoid carcinoma (MEC) is the most frequently diagnosed tumor of all malignant salivary gland neoplasms, and given its rarity, many clinical details about MEC remain poorly studied and understood [5,6]. MEC has historically displayed pathologic, biological, and clinical variability. Prior research has demonstrated that the histopathologic grade and clinical stage strongly correlate with prognosis [7].

MEC exhibits variable clinical presentation in both pediatric and adult populations, as mentioned in previous case reports and limited case series [8]. The current study examined a large cohort of pediatric and adult MEC patients to identify demographic, pathologic, and clinical factors that may impact patient outcomes and could be used to categorize MEC patients for proper treatment selection and clinical trial enrollment in the future.

## 2. Materials & Methods

The Surveillance, Epidemiology and End Results (SEER) database initiated by the National Cancer Institute in 1973 covers approximately 28% of the U.S. population. The SEER*Stat software (Version 8.4.0) (https://seer.cancer.gov/seerstat/, accessed on 25 October 2022). was used to collect data from 2000 to 2018 using the International Classification of Diseases version 3 (ICD-O-3) and anatomical code (8330/3) and histological codes were C07.9, C08.0, C08.1, C08.8, and C08.9. The data was exported to Statistical Analysis System (SAS) (SAS/ACCESS^®^ 9.4 Interface to ADABAS: Reference, SAS Institute Inc 2013, Cary, NC, USA) for Kaplan Meier graphs and rest of the analysis.

Demographic and clinical data included age, race, tumor grade, tumor size, lymph node status, metastasis, surgical treatment, radiotherapy, chemotherapy, overall survival, survival with surgery, survival with radiation therapy and, survival with chemotherapy. The cases included were ‘microscopically confirmed “positive histology”, positive exfoliative cytology, positive histology, immunophenotyping and/or positive genetic studies, and positive microscopic confirm, method not specified. Exclusion criteria included patients without histological confirmation, those diagnosed with in situ cancers, alive with no survival time, and with diagnosis made through death certificates and/or autopsy only. Studied variables included age, sex, race, tumor stage, and type of treatment received (surgery, radiation, chemotherapy, various combinations of the three, or unknown/no treatment). Children were defined as those aged ≤ 18 years while adults aged > 18 years. The endpoints examined included overall survival, mortality, and 1-, 2-, 3-, 4-, and 5-year cancer-specific survival rates. The 5 years were considered the endpoint of the study. 

This study used the cox regression method to calculate hazard ratios and identify the independent factors that affect survival. Data that were either unidentified or missing were removed from multivariate analysis. Univariate analysis was performed to identify significant factors for the multivariate model with an accepted *p*-value of 0.25. Multivariate cox regression analysis was used to analyze the data, statistical significance was defined as *p* < 0.05.

## 3. Results

### 3.1. Demographical Characteristics

Of the entire cohort, 3.7% (*n* = 165) were in the pediatric group and 96.3% (*n* = 4342) were in the adult group. In both the pediatric and adult groups, the majority of patients were women (51.5% and 52.2%, respectively). The pediatric group had a higher proportion of the Black Americans affected (17.6%) compared to the adult counterpart (12.7%). Conversely, a higher proportion of Whites (75.4%) and American Indian/Alaska Natives (6.9%) were found in the adult group compared to the pediatric group (72.1% and 0.6%, respectively) (Table 1). 

### 3.2. Tumor Characteristics

The overall most common histology was moderately differentiated MEC carcinoma (39.2%). This was true in both the pediatric and adult group (47.9% and 38.9%, respectively). However, the amount (*n*) of pediatric patients with a well differentiated MEC carcinoma was significantly greater than in their adult counterparts (*p* < 0.05). Additionally, adults had more poorly differentiated disease (11.3%) and metastatic disease (9.3%) compared to their pediatric counterparts (3.0% and 4.8%, respectively) (*p* < 0.05). All microscopic tumors were found in the adult age group (*n* = 15), making up 0.3% of total cases. There were statistically greater amounts of tumors less than 2 cm in size found in adults compared to the pediatric group (*p* < 0.05). Conversely, the pediatric group had statistically significant tumors between 2 and 4 cm in size (*p* < 0.05). Overall, most of the staging was localized (58.8%) (Table 2).

### 3.3. Treatment Characteristics:

**Figure 1:**
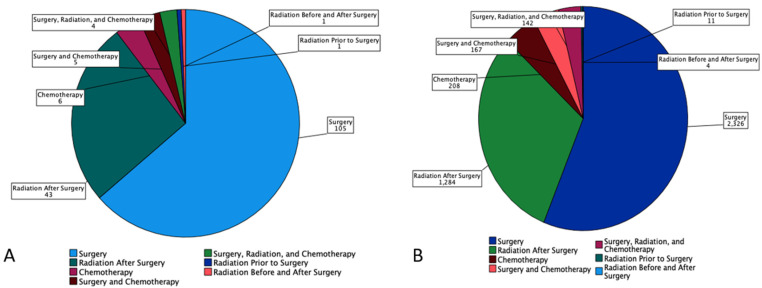
Pie chart based on 4507 Patients with Mucoepidermoid Carcinoma (MEC) from SEER Database (2000–2018). It displays number of patients (*n*) undergoing each treatment modalities (surgery, radiation prior to surgery, radiation before and after surgery, radiation after surgery, chemotherapy, surgery and chemotherapy, and combination of surgery, radiation, and chemotherapy by (**A**) Pediatrics and (**B**) Adults.

**Figure 2:**
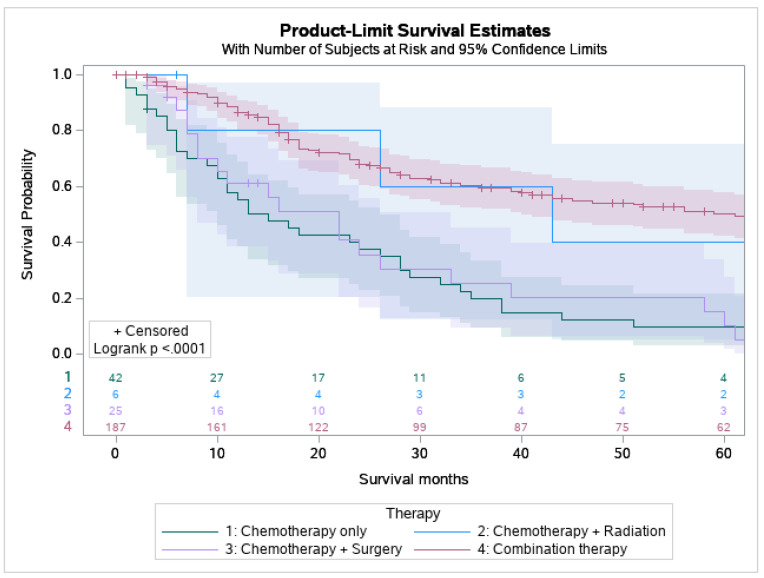
Kaplan Meier graph displaying survival analysis of patients with mucoepidermoid carcinoma (MEC) over the span of 60 months by various treatment modalities involving chemotherapy including chemotherapy only, chemotherapy and radiation, chemotherapy and surgery, and combination of surgery, chemotherapy, and radiation.

53.9% of MEC patients underwent surgery, and 29.8% underwent combination surgery and radiotherapy. Only 4.7% of patients underwent chemotherapy. 63.6% of the pediatric patients underwent surgery, which is statistically more than the 53.5 % of the adult patients who underwent surgery (*p* < 0.05) (Figure 1). A combination of surgery and radiation was used more frequently in adults (30.0%) than in the pediatric population (27.3%) (Table 3). The lowest overall survival was observed in patients who received chemotherapy only, whereas patients who are treated with combination therapies, i.e., surgery, chemotherapy, and adjuvant radiation had the best overall survival (Figure 2).

### 3.4. Survival Characteristics by Age

**Figure 3:**
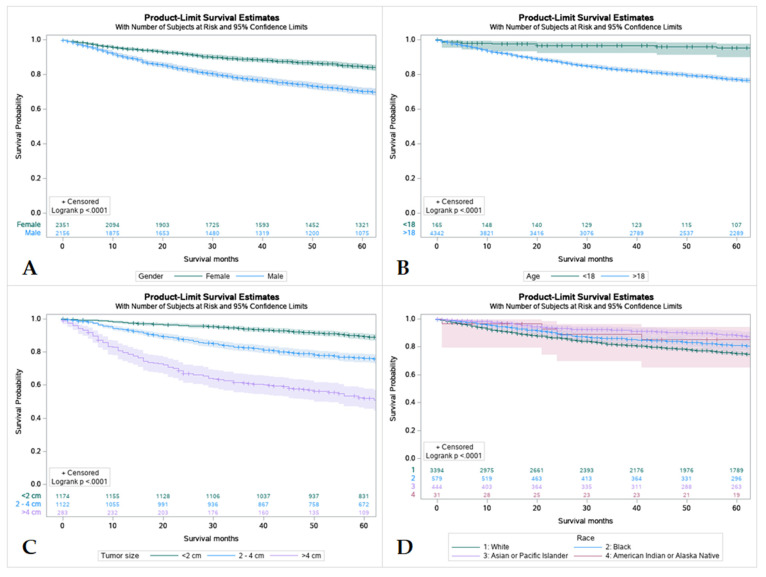
Kaplan Meier graphs displaying comparative survival analysis of patients with mucoepidermoid carcinoma (MEC) over the span of 60 months (5 years) by (**A**) Gender, (**B**) Age, (**C**) Tumor size, and (**D**) Race. Females had a high 5-year survival compared to males. Patients < 18 years old had higher 5-year survival compared to adult (>18 years old). Whites had the worst 5-year survival compared to Blacks, Asian or Pacific Islanders, and American Indian or Alaska Native. Patients with tumors less than 2 cm in size had the highest 5-year survival and patients with tumors less than 4 cm had the lowest.

**Figure 4:**
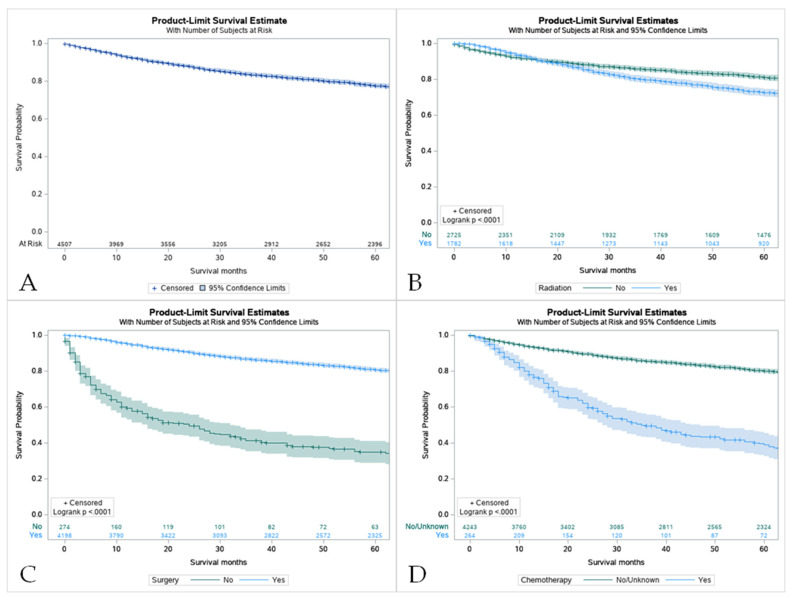
Kaplan Meier graphs displaying comparative survival analysis of patients with mucoepidermoid carcinoma (MEC) over the span of 60 months (5 years) by (**A**) Overall survival, (**B**) Survival with radiation therapy, (**C**) Survival with surgery, and (**D**) Survival with chemotherapy. Patients with MEC who did not receive radiation or chemotherapy had higher 5-year survival compared to patients who did receive one or the other. Comparatively, patients who underwent surgery had a statistically significant higher 5-year survival compared to those who did not (*p* < 0.05).

The 5-year survival rate for all of the patients with MEC was 86.8%. Overall, pediatric patients had a higher overall survival rate in comparison to their adult counterparts (8.0 vs. 6.6 years) (Figure 3B). For pediatric patients, the 5-year survival rate was 100%. The same rate was found in pediatric groups that received only surgery and some variation in radiation and surgery (100%). Across all treatment modalities, the pediatric group displayed higher survival rates (Table 3). Surgery offered the best approach to improve survival in both patient groups (Figure 4C). It was noticed that the use of surgery as the primary treatment option significantly increased the overall survival (OS) in the pediatric population (8.0 ± 5.8 years) compared to adults (7.2 ± 5.3 years; *p* < 0.001). Adults that received radiation before and after surgery had the worst 5-year survival rate at 37.5%. However, chemotherapy displayed the lowest overall survival (40.8%, Confidence Interval (CI) 33.5–47.9) with patients who had not received this treatment having higher survival rates (Table S1, Figure 4D). A combination of surgery, chemotherapy, and radiation offered no survival benefit in adults with 0% surviving after 5 years when radiation was given before and after surgery (Table S2). 

Children had a significantly lower overall mortality (5.5%) than adults (28.6%), as well as higher 1- and 5-year cancer-specific survival rates (100% and 97.5% vs. 100% and 89.1%, respectively) (Table S3).

### 3.5. Survival by Sex, Age, Tumor Size and Race

Females had a higher 5-year survival rate compared to males (86.5%, and 77%, respectively). Survival rates were found to have an inverse relationship with tumor size with tumors less than 2 cm having the highest survival rate and tumors larger than 4 cm having the lowest. Out of all patients, Caucasians had the worst 5-year survival rate at only 77.7% compared to African Americans and American Indians, Asians, and Pacific Islanders (83.2%, and 89.4%, respectively) (Table S3 and Figure 3).

### 3.6. Survival by Tumor Stage, Grading and Metastasis at the Time of Diagnosis

**Figure 5:**
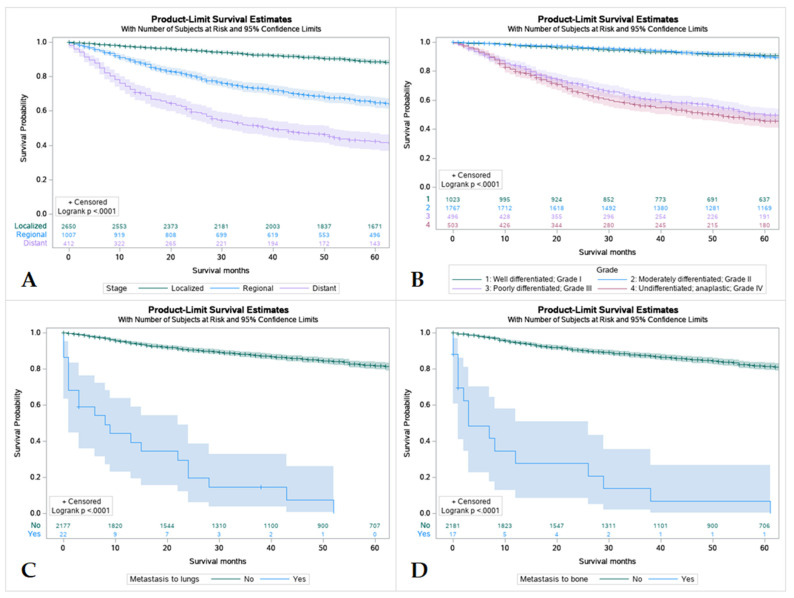
Kaplan Meier graphs displaying comparative survival analysis of 4507 patients with mucoepidermoid carcinoma (MEC) over the span of 60 months (5 years) by (**A**) Tumor Stage, (**B**) Tumor grade, (**C**) Metastasis to the lung, and (**D**) Metastasis to the bone. Patients with localized tumor staging had higher 5-year survival than regional or distant spread. Well-differentiated (Grade I) and moderately differentiated (Grade II) tumors were found to have higher 5-year survival compared to poorly differentiated or undifferentiated tumors (*p* < 0.005). Metastasis to lungs or bone were found to mean statistically lower 5-year survival in patients compared to those without metastasis.

Overall, patients with tumors that were localized had better survival rates in comparison to regional and distant spread, with distant spread having the lowest among the three. Patients with tumors that were well differentiated or moderately differentiated had better survival outcomes in comparison to patients with poorly differentiated or undifferentiated tumors. Patients without metastasis were found to have higher rates of survival over 5 years regardless of location (Figure 5).

### 3.7. Multivariate Analysis

ANOVA (analysis of variance) was performed on the variables (age, race, gender, tumor size, tumor stage, treatment modalities, extent of disease) to identify the significant factors used for cox regression model with significance set at *p* < 0.25. Hazard ratio and confidence intervals were documented with significance set at *p* < 0.05. Cox Regression analysis revealed that being an adult (HR 7.4, CI 1.8–29.7), male (HR 0.8; CI 0.7–1.0), Asian or Pacific Islander race (HR 0.5; CI 0.3–0.8), poorly differentiated (HR 3.8; CI 2.6–5.6), undifferentiated grade (HR 4.5; CI 3.1–6.4), the regional extension of the disease (HR 2.1; CI 1.7–2.7), and distant extent of disease (HR 3.2; CI 2.4–4.2) were related to increased mortality (Table 4). 

## 4. Discussion

MEC is the most frequently diagnosed malignancy of the salivary glands in both children and adults. This study demonstrates different characteristics and outcomes between adult and pediatric patients with MEC. Several small-scale studies in the past have described the gender distribution of MEC. Most of them support the male preponderance among adult cases of MEC and describe that MEC in male patients has a worse prognosis [1,3,5]. However, the pediatric gender distribution is less well-defined [2,4]. Interestingly, contrary to our findings, prior studies on MEC in children have shown a slight predilection for the male sex [6]. There have been other studies of pediatric salivary malignancies that have shown a female preponderance, although these studies did not report statistics specifically for MEC [7].

The grade of differentiation plays a crucial role in the long-term outcomes of oncology patients. As mentioned in our study, more patients with poorly differentiated cancer were observed in the adult group, and our findings were supported by the studies published so far [8]. Sultan et al. found in their retrospective database study comparing adult and pediatric cases of MEC that children had more favorable features, with most tumors being localized without extension to adjacent tissues or lymphatic spread (76% vs. 50% in adults, *p* < 0.001). They also found that most tumors in children were well-differentiated or moderately differentiated (88% vs. 49% in adults, *p* < 0.001). The same study showed better survival among pediatric cases of MEC compared to adults with 5-year overall survival for children of 95% ± 1.5%, compared with 59% ± 0.5% for adults (*p* < 0.001), which is similar to our findings [9].

Moreover, Kupferman et al. specifically looked at the outcomes and treatment complications of pediatric patients with malignancies of the salivary glands. They reported that the pediatric population had better survival rates and that overall survival depends on the level of differentiation, the status of margin involvement after surgical resection, and neurovascular invasion [10]. A study by Yamazaki et al. supports this correlation. Using multivariate analysis, they analyzed the clinicopathological features of 45 patients with MEC and found that in addition to tumor (T) and node (*n*) classification, age had a statistically significant association with survival (*p* = 0.04; hazard ratio = 0.09), with older age conferring a worse prognosis [11]. Another investigation of 119 patients with MEC by Li et al. demonstrated that in addition to tumor grade and stage, the clinicopathological parameter that correlated with lower survival was age > 40 years (*p* < 0.05) [12].

In addition to age, tumor size also plays a critical role in outcomes. High-grade and advanced-stage tumors are well-recognized predictors of poor outcomes. Liu et al. reported that overall disease-free survival at five years was 80.74%, with 98.0% for low-grade tumors, 86.5% for intermediate-grade tumors, and 38.5% for high-grade tumors (*p* < 0.001) [13]. Likewise, Ali et al. and Chen et al. analyzed the cause-specific mortality and demographical distribution of MEC and found that the disease-specific survival (DSS) rate was significantly lower for high-grade MEC than for all other grades [14,15].

The correlation between tumor grade and overall prognosis has been reported at least a decade ago. To establish this relationship, McHugh et al., in 2011, in a retrospective study, reported that tumors with low to intermediate differentiation have a better overall survival rate compared to the high-grade variant. They also reported that a higher age at diagnosis and perineural involvement at the time of diagnosis are associated with poor prognoses [16]. Their results indicate that both low-and intermediate-grade MECs have comparable and favorable survival, suggesting a similar management approach, whereas high-grade and advanced-stage disease necessitates aggressive treatment plans to improve outcomes. These results support our findings that advanced tumor and histological grade are critical prognostic factors for MEC. 

In the current study, most patients with MEC underwent surgery, with 41% receiving a combination of surgery and radiotherapy. Analysis by age showed that more children underwent primary surgical resection than adults (63.6% vs. 53.5%), *p* < 0.05. Moreover, surgical resection significantly improved overall survival in children as compared to adults (34.9 ± 0.5 vs. 22.6 ± 0.5 years; *p* < 0.001) [17]. These results could be a result of the fact that more children with MEC have a lower grade and less advanced tumors than adults, thus more likely to undergo primary surgical resection [18].

The type of therapy determines the patient prognosis. Primary surgical resection is the mainstay of treatment, and efforts have been made to supplant primary surgical resection with radiation therapy. In a retrospective case review of 61 pediatric patients with major salivary gland malignancies, Kupferman et al. reported that the majority of patients underwent surgical resection (75%), while 45% also received external beam radiation. They found a survival rate of 93% at 5 years with 26% developing recurrence. Their results demonstrate that although the mainstay of treatment for pediatric MEC is surgical resection, there is a benefit of adding radiation therapy to patients, especially with high-grade advanced tumors to prevent locoregional recurrence [6].

Chen et al. investigated the outcomes of patients with localized MEC who received surgery and postoperative radiation. They found higher treatment failure rates, particularly in high-grade and advanced tumor stages [19]. They also noticed that the higher the tumor stage, the more metastatic potential the tumor had. These findings point to the critical interplay between tumor grade and stage, prognosis, and treatment selection in MEC. Ali et al. noted in their study of cause-specific mortality in patients with MEC that the cause of death in the majority of patients was distant metastases rather than locoregional recurrence [14]. This highlights the need to explore systemic treatments such as chemotherapeutics, immunotherapeutics and targeted agents in MEC treatment to improve the prognosis of patients with advanced disease.

### 4.1. Genomic Alterations, Ongoing Investigations, and Insights in Future Therapeutic Approaches

**Figure 6:**
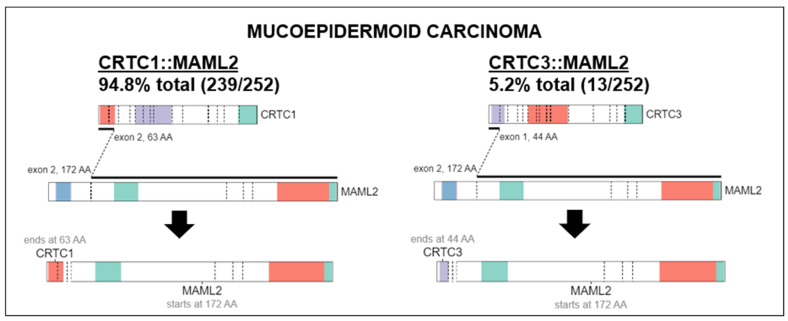
Characteristic fusions identified in MEC. MAML2 fusion to CRTC1 (**Left**) was found in 94.8% of all cancers histologically classified as MEC while MAML2 fusion to CRTC3 (**Right**) was found in the remaining 5.2%. Fusion data were visualized using ProteinPaint software (https://pecan.stjude.cloud/proteinpaint, accessed on 15 October 2022).

MECs have a distinct molecular prolife from other salivary gland carcinomas. Translocation, t(11;19) resulting in fusion CTRC1-MAML2 oncogene that acts as a transcription factor on Notch and CREB regulatory pathways is present in 56–88% of MECs [20,21,22]. CRTC1-MAML2 translocations occur more frequently in low and intermediate-grade tumors [23]. Although useful for diagnosis, it does not appear to be a strong prognostic marker [21,24]. A comprehensive genomic profiling study of MEC revealed 183 genomic alterations in 80 unique genes [25]. The commonly detected alterations were those involving *TP53* (41.7%) and *CDKN2A* (41.6%). Other genes altered in ≥10% of cases included *CDKN2B* (29.2%), *BAP1* (20.8%), *PIK3CA* (20.8%), *HRAS* (10.4%) and *BRCA1/2* (10.5%). Alterations in the fibroblast growth factor pathway were seen in 12.5% (6.3% *FGFR1*, 2.1% each *FGF3*, *FGF4*, *FGF19*). *NOTCH1/2* and *NF1* alterations comprised 4.3% each. *HER2* gene (*ERBB2*) amplification was seen in 8.3% cases which is similar to another study that showed 14.3% by immunohistochemistry (IHC) and fluorescent in-site hybridization (FISH) [25,26]. However, higher levels of HER2 overexpression has been reported in larger seminal studies, viz. 38% by IHC and 21% gene amplification by FISH [27]. High tumor mutational burden (>10 mutations/megabase) was present in 10% of cases [25]. High-grade tumors had more genomic alterations, higher frequency of *TP53* alterations and PI3K/mTOR pathway activation, including PI3KCA mutations than low- or intermediate-grade tumors [22,25]. IHC study of MEC showed high-level cell surface expression of *EGFR* (45.5%) and *MUC1* (44%) [28]. In addition of *HER2*, which is a marker of worse prognosis, IHC can be used to detect PCP4/PEP19 which is a good prognosis marker [29]. Activation of the EGFR pathway frequently through increased gene copy number of *EGFR* or *ERBB2* is correlated with the development of high-grade MEC and appears to be independent of CTCR1-MAML2 translocation [26,30]. Our study found that in 94.8% of histologically classified MEC, there was a MAML2 fusion to CRTC1 while the remaining 5.2% contained a MAML2 fusion with CRTC3 (Figure 1). This was visualized using ProteinPaint, an online application that displays genetic lesions such as gene fusions along with RNA expression [31]. ProteinPaint also provided a cross-study comparison with various research studies, specifically pediatric cancer data with adult data [31]. While there was only two fusions found for MEC on COSMIC (https://cancer.sanger.ac.uk/cosmic, accessed on 15 October 2022), the CRTC1::MAML2 alteration was also documented in 2 hidradenomas, 11 adenolymphomas (also known as Warthin tumors), 1 rare case of malignant Warthin tumor (Figure 6).

Although rare, reports of MECs in germline *BAP1* and *BRCA* variants may hint those alterations in these genes play a role in pathogenesis [32,33]. A messenger RNA study of 28 extracellular matrix-related genes in salivary gland carcinomas, showed overexpression of 10 genes (36%) in MECs, which correlates with the commonly noted clinical finding of desmoplastic stromal reaction in MECs [34,35]. MEC, displays a partial squamoid differentiation overexpress *LAMB3*, which is a part of laminin 332 and associated with adverse outcomes in several primaries [34]. At least two-thirds of salivary duct carcinomas express androgen receptors (AR) and this can be exploited therapeutically [36,37]. However, AR expression is rare in MEC [38,39]. *NTRK* gene fusions are less common in MECs than secretory carcinomas [40]. Programmed death-1 ligand-1 (PD-L1) positivity in tumor cell membrane was detected in 6 out of 11 (63.6%) MEC patients [41].

The rarity of salivary gland carcinomas deters large-scale commercial interest for drug development. Despite being the most common malignant salivary gland tumors, MECs do not justify trials solely dedicated to this entity. There is no drug specifically approved for MEC or for that matter any salivary gland carcinomas. There are 34 active clinical trials registered at clinicaltrials.gov (accessed on 14 September 2022) enrolling patients with salivary gland carcinomas and the ones that have MECs specifically listed are outlined in Table 4. However, none of these are exclusive to MECs. Recently, the ease and availability of broad next-generation sequencing (NGS) panels have ushered in the era of targeted approaches for salivary gland carcinomas including MECs [42]. A study utilizing 468 gene NGS panels in 125 patients with recurrent or metastatic salivary gland cancers identified actionable alterations in 33% of patients [43]. When present, NTRK inhibitors and androgen deprivation therapy can effectively target *NTRK* fusion and AR-expressing MECs, respectively. Anti-HER2-directed therapies such as trastuzumab with chemotherapy and ado-trastuzumab emtansine are commonly used off-label in *HER2* overexpressing and/or overamplified MECs [44,45]. Due to the high-level expression of *EGFR*, anti-EGFR antibodies such as cetuximab held theoretical promise as a component of systemic therapy for advanced disease [46]. In spite of case reports of the benefit of cetuximab in MEC, a phase II trial evaluating cetuximab as a component of systemic therapy for unselected recurrent/metastatic salivary gland carcinomas was negative further highlighting the need for biomarker-driven treatments [47,48]. The farnesyltransferase inhibitor tipifarnib showed 58% stable disease as the best response in HRAS mutated patients with salivary gland carcinoma cohort which comprised 8% MECs [49]. A phase 1b study of pembrolizumab for PD-L1 positive salivary gland carcinomas that comprised 12% of MECs reported an objective response rate of 12% (no complete response) [50]. For fit patients requiring systemic treatment and devoid of a target, a platinum and anthracycline-based regimen, commonly cisplatin, doxorubicin, and cyclophosphamide (CAP) has the highest level of evidence [51]. Various agents and strategies aimed at the MYB-NFIB pathway; NOTCH 1, 2, 3; PRMT-5, histone deacetylation; KIT/VEGFR and ^111^Lutitium-PSMA are under investigation [52]. The ongoing clinical trials from National Institute of Health (NIH) enrolling patients of mucoepidermoid carcinoma (https://clinicaltrials.gov/, accessed on 14 September 2022) are listed in (Table 5). 

### 4.2. Limitations

Our study attempts to describe the clinical and demographic aspects of MEC patients using a large national database. Our study has limitations shared with most of the database studies. First, critical clinical factors such as socioeconomic factors, mitotic tumor index, and associated endocrine pathologies that might affect the interpretation of results, are not coded correctly in the SEER database. Second, the treatment course and stage-specific management were missing from the SEER database. In addition, the type of chemotherapy, radiation dosing and scheme, and side effects of the various modalities are not available in the SEER database.

## 5. Conclusions

Mucoepidermoid carcinoma is an aggressive salivary gland neoplasm predominantly found in Caucasians and is more common in adults. Surgical resection of MEC is more common in children, which significantly improves overall survival. More adults received combination therapy with surgery and postoperative radiation, corresponding to the increased incidence of advanced disease among adults with MEC. Older age, tumor size > 2 cm, poor or undifferentiated grade, and regional disease stage were associated with increased mortality. Accordingly, adult patients, those with high-grade or advanced tumors, or other prognostic factors positively correlated with disease progression, should be evaluated for surgical resection with postoperative radiotherapy, with consideration for chemotherapy as appropriate. Based on the results of our data, we also anticipate that clinicians and other healthcare providers can better counsel their patients about the short- and long-term prognosis of salivary gland mucoepidermoid carcinoma both in pediatric and adult populations.

## Figures and Tables

**Table 1:** Demographics and Clinical Profile of 4507 Patients with Mucoepidermoid Carcinoma (MEC) from the Surveillance Epidemiology and End Result (SEER) Database (2000–2018). Patients are divided into two main groups: pediatric (≤18 years old) and adult (>18 years old). The overall survival, gender differences and racial disparities are compared both in pediatric and adult groups.

	Overall	Age ≤ 18	Age > 18
*n* (%)	4507 (100.0)	165 (3.7)	4342 (96.3)
Age (Mean ± SD)	55.1 ± 18.9	13.5 ± 3.6	56.6 ± 17.4
Gender (%)			
Male	2156 (47.9)	80 (48.5)	2076 (47.8)
Female	2351 (52.1)	85 (51.5)	2266 (52.2)
Race *n* (%)			
White	3394 (75.3)	119 (72.1)	3275 (75.4)
Black	579 (12.8)	29 (17.6)	550 (12.7)
Asian or Pacific Islander	444 (9.9)	14 (8.5)	430 (9.9)
American Indian/Alaska Native	31 (0.7)	1 (0.6)	30 (6.9)
Unknown	59 (1.3)	2 (1.2)	57 (1.3)

Abbreviations: *n* = number; SD = standard deviation.

**Table 2:** Tumor Characteristics and lymph node involvement of 4507 Patients with Mucoepidermoid Carcinoma (MEC) from the Surveillance Epidemiology and End Result (SEER) Database (2000–2018). Tumor grade, tumor stage, tumor size, and lymph node involvement are compared between pediatric and adult groups. Chi-Square test analysis was performed to determine significant differences between age groups.

	Overall	Age ≤ 18	Age > 18
*n* (%)	4507 (100.0)	165 (3.7)	4342 (96.3)
Grade *n* (%)			
Well-differentiated	1023 (22.6)	52 (31.2) *	971 (22.4) *
Moderately differentiated	1767 (39.2)	79 (47.9)	1688 (38.9)
Poorly differentiated	496 (11.0)	5 (3.0) *	491 (11.3) *
Undifferentiated	503 (11.2)	10 (6.1) *	493 (11.4) *
Unknown	429 (9.5)	14 (8.5)	415 (9.6)
Stage *n* (%)			
Localized	2650 (58.8)	99 (60.0)	2551 (58.8)
Regional	1007 (22.3)	49 (29.7) *	958 (22.1) *
Distant	412 (9.1)	8 (4.8) *	404 (9.3) *
Unstaged	149 (3.3)	4 (2.4)	145 (3.3)
Tumor Size			
Microscopic	15 (0.3)	0 (0.0)	15 (0.3)
<2cm	1363 (30.2)	41 (24.8) *	1322 (30.4) *
2–4 cm	924 (20.5)	47 (28.5) *	877 (20.2) *
>4cm	282 (6.3)	15 (0.9)	267 (6.1)
Lymph Node Involvement *n* (%)			
Yes	739 (16.4)	25 (15.1)	714 (16.4)
No	2153 (47.8)	101 (61.2)	2052 (47.3)

* Signifies statistically significant difference found between pediatric and adult groups for a given variable, defined as *p* < 0.05. Abbreviations: *n*, number; SD, standard deviation.

**Table 3:** Treatment modalities of 4507 Patients with Mucoepidermoid Carcinoma (MEC) from SEER Database (2000–2018) are first shown. This is divided into pediatric (≤18 years old) and adult groups (>18 years old). Survival length in years based on treatment type (surgery, radiation, combination of radiation and surgery in various orders, chemotherapy, surgery and chemotherapy, and combination of all 3 types). Chi-Square test analysis was performed to determine significant differences between age groups.

	Overall	Age ≤ 18	Age > 18
*n* (%)	4507 (100.0)	165 (3.7)	4342 (96.3)
Treatment *n* (%)			
Surgery Only	2431 (53.9)	105 (63.6) *	2326 (53.5) *
Radiation Prior to Surgery	12 (0.3)	1 (0.6)	11 (0.3)
Radiation After Surgery	1327 (29.4)	43 (26.1)	1284 (29.6)
Radiation Before and After Surgery	5 (0.1)	1 (0.6)	4 (0.1)
Chemotherapy	214 (4.7)	6 (3.6)	208 (4.8)
Combined Surgery and Chemotherapy	172 (3.8)	5 (3.0)	167 (3.8)
Combination of Surgery, Radiation, Chemotherapy	146 (3.2)	4 (2.4)	142 (3.3)
Survival by treatment (years ± SD)			
Surgery Only	7.3 ± 5.4	8 ± 5.8	7.2 ± 5.3
Radiation Prior to Surgery	5.8 ± 5.7	15.9	4.2 ± 3.8
Radiation After Surgery	6.8 ± 5.1	8.1 ± 4.6	6.8 ± 5.1
Radiation Before and After Surgery	5.5 ± 4.4	7.6	5.0 ± 4.8
Chemotherapy	2.1 ± 2.4	0.9	2.1 ± 2.4
Combined Surgery and Chemotherapy	4.4 ± 4.1	7.8 ± 5.6	4.0 ± 3.9
Combination of Surgery, Radiation, Chemotherapy	4.4 ± 4.1	9.2 ± 4.8	4.2 ± 4.0
Unknown	3.1 ± 4.3	0.6 ± 1.0	3.1 ± 4.3
Overall Mortality *n* (%)	1252 (27.8)	9 (5.5) ***	1243 (28.6)
Cancer-Specific Mortality *n* (%)	569 (12.6)	5 (3.0) ***	564 (13.0)

Abbreviations: *n*, number; SD, standard deviation; *, statistically significant difference between pediatric and adult patients for a given variable, defined as * *p* < 0.05, *** *p* < 0.001.

**Table 4:** Cox Regression analysis of age, sex, race, tumor grade, and tumor staging influencing mortality in 4507 patients with Mucoepidermoid Carcinoma (MEC) from the Surveillance Epidemiology and End Result (SEER) Database (2000–2018).

Variables	Univariate Analysis	Multivariate Analysis	
	ANOVA F Value (*p*-Value)	Hazard Ratio (*p*-Value)	Confidence Interval (CI)
Age > 18	6.1 (0.014)	7.4 (0.005)	1.8–29.7
Male Gender	19.5 (<0.001)	0.8 (0.04)	0.7–1.0
Asian or Pacific Islander Race	2.4 (0.063)	0.5 (0.001)	0.3–0.8
Poorly differentiated Grade	124.6 (<0.001)	3.8 (<0.001)	2.6–5.6
Undifferentiated Grade		4.5 (<0.001)	3.1–6.4
Regional Extent of Disease	129.4 (<0.001)	2.1 (<0.001)	1.7–2.7
Distant Extent of Disease		3.2 (<0.001)	2.4–4.2

**Table 5:** Selected ongoing clinical trials enrolling patients with mucoepidermoid carcinoma (Source: https://clinicaltrials.gov/, accessed on 14 September 2022).

Trial Number (Name)	Study Title	Study Type	Study Arms	Primary Outcome	Status (on 09/14/2022)
NCT01473784	Transoral robotic surgery in treating patients with benign or malignant tumors of the head and neck	Single arm, open label	Transoral robotic surgery (TORS) using the Da Vinci robotic surgical system	Feasibility of TORS	Recruiting
NCT01586767	Intensity-modulated or proton radiation therapy for sinonasal malignancy	Phase II, non-randomized	Proton beam therapy vs. IMRT	Local control rate at 2 years	Recruiting
NCT04249947	P-PSMA-101 CAR-T Cells in the treatment of subjects with metastatic castration-resistant prostate cancer (mCRPC) and advanced salivary gland cancers (SGC)	Phase II, non-randomized	P-PSMA-101 CAR-T cells single and multiple doses following conditioning regimen	Safety, maximum tolerated dose and efficacy	Recruiting
NCT03602079	Study of A166 in patients with relapsed/refractory cancers expressing HER2 antigen or having amplified HER2 gene	Phase I–II, non-randomized	Phase I: Six dose levels Phase II: Treatment with A166 with recommended phase II dose	Maximum tolerated dose, number of patients with dose limiting toxicities	Active, not recruiting
NCT00003251	Amifostine plus chemotherapy and radiation therapy in treating patients with advanced, unresectable head and neck cancer	Phase I/II	N/A	N/A	Unknown *
NCT01220583	Radiation therapy with or without chemotherapy in treating patients with high-risk malignant salivary gland tumors that have been removed by surgery	Phase II/III, randomized	3D-CRT or IMRT vs. 3D-CRT or IMRT with cisplatin	PFS	Active, not recruiting

Abbreviations; IMRT, intensity-modulated radiation therapy; P-PSMA-101 CAR-T; prostate-specific membrane antigen-specific Centyrin-based chimeric antigen receptor T; A166, antibody–drug conjugate targeting HER2 expressing cancer cells; N/A, not available; PFS, progression-free survival; 3D-CRT, 3-dimensional conformal radiotherapy. * Last update posted in December 2013.

## Data Availability

All data are publicly available and will be provided upon appropriate request from the corresponding author.

## Supplementary Materials

The following supporting information can be downloaded at: https://www.mdpi.com/article/10.3390/cancers15010250/s1, Table S1: Survival data of 4507 Patients with Mucoepidermoid Carcinoma (MEC) from the Surveillance Epidemiology and End Result (SEER) Database (2000–2018); Table S2: Survival data of 4507 Patients with Mucoepidermoid Carcinoma (MEC) from the Surveillance Ep-idemiology and End Result (SEER) Database for Combination Therapy.(2000–2018); Table S3: Survival data of 4507 Patients with Mucoepidermoid Carcinoma (MEC) from the Surveillance Ep-idemiology and End Result (SEER) Database by Demographics (2000–2018).
