# Supplementary material for: Mucoepidermoid Carcinoma of the Salivary Gland: Demographics and Comparative Analysis in U.S. Children and Adults with Future Perspective of Management
Source: Cancers (Basel). 2022 Dec 30;15(1):250. doi: 10.3390/cancers15010250 (PMC9818327; doi:10.3390/cancers15010250)
Supplement: Supplementary file 1 [file cancers-15-00250-s001.zip › cancers-2090141-supplementary.pdf]

cancers

MDPI

Supplementary Materials

Mucoepidermoid Carcinoma of the Salivary Gland: Demographics and Comparative Analysis in Children and Adults with an Insight into the Future Perspective of Management: A U.S. Population-Based Study

Asad Ullah, Jaffar Khan, Abdul Waheed, Nabin Raj Karki, Mya Goodbee, Abdul Qahar Khan Yasinzai, Bisma Tareen, Agha Wali, Khaleel Ahmad Khan, Muhammad Samsoor Zarak, Israr Khan, Andrea Agualimpia Garcia, Adil Khan, Marjan Khan, Sana Jogezai, Junaid Ahmad, Luis Velasquez Zarate, Nikhil Patel, Nagla Abdel Karim and Saleh Heneidi

Table S1. Survival data of 4507 Patients with Mucoepidermoid Carcinoma (MEC) from the Surveillance Epidemiology and End Result (SEER) Database (2000–2018).

**Overall Cumulative Survival (%, 95% Confidence Interval)**			
1 year	93.6 (92.7–94.4)		
2 years	89.0 (87.8–90.1)		
3 years	84.8 (83.4–86.0)		
4 years	82.4 (80.9–83.7)		
5 years	79.8 (78.2–81.2)		
Disease-specific survival			
	Overall	Age ≤18	Age >18
1 year	95.4 (94.6–96.1)	100.0	97.5 (95.2–98.7)
2 years	92.1 (91.1–93.0)	100.0	93.9 (90.7–96.0)
3 years	89.3 (88.1–90.4)	100.0	91.6 (88.1–94.1)
4 years	88.1 (86.9–89.3)	100.0	89.8 (86.0–92.7)
5 years	86.8 (85.5–88.0)	100.0	89.1 (85.1–92.1)
Surgery Only Survival			
	Overall	Age ≤18	Age >18
1 year	95.8 (95.0–96.5)	99.2 (94.7–99.9)	97.1 (96.4–97.6)
2 years	91.5 (90.4–92.5)	98.4 (93.7–99.6)	93.8 (92.8–94.7)
3 years	87.6 (86.3–88.8)	98.4 (93.7–99.6)	91.2 (90.1–92.3)
4 years	85.2 (83.8–86.5)	98.4 (93.7–99.6)	90.1 (88.8–91.2)
5 years	82.6 (81.1–84.1)	98.4 (93.7–99.6)	88.8 (87.5–90.0)
Radiation Prior To Surgery Survival			
	Overall	Age ≤18	Age >18
1 year	66.7 (33.7–86.0)	100.0	72.2 (36.1–90.1)
2 years	58.3 (27.0–80.1)	100.0	61.9 (27.2–83.8)
3 years	58.3 (27.0–80.1)	100.0	61.9 (27.2–83.8)
4 years	58.3 (27.0–80.1)	100.0	61.9 (27.2–83.8)
5 years	48.6 (19.2–73.0)	100.0	49.5 (17.0–75.6)
Radiation After Surgery Survival			
	Overall	Age ≤ 18	Age >18
1 year	94.9 (93.5–96.0)	100.0	95.7 (94.4–96.7)
2 years	87.9 (85.9–89.7)	100.0	90.3 (88.5–91.9)
3 years	81.5 (79.2–83.7)	100.0	85.7 (83.6–87.6)
4 years	78.3 (75.8–80.6)	100.0	83.6 (81.3–85.6)

5 years	74.1 (71.4–76.6)	100.0	81.2 (78.8–83.4)
Radiation Before and After Surgery Survival			
	Overall	Age ≤18	Age >18
1 year	80.0 (20.4–96.9)	100.0	75.0 (12.8–96.1)
2 years	80.0 (20.4–96.9)	100.0	75.0 (12.8–96.1)
3 years	80.0 (20.4–96.9)	100.0	75.0 (12.8–96.1)
4 years	80.0 (20.4–96.9)	100.0	75.0 (12.8–96.1)
5 years	53.3 (6.8–86.3)	100.0	37.5 (1.1–80.8)
Chemotherapy Survival			
	Overall	Age ≤18	Age >18
1 year	78.4 (72.0–83.5)	80.0 (20.4–96.9)	81.1 (74.9–86.0)
2 years	62.5 (55.3–68.9)	80.0 (20.4–96.9)	65.8 (58.5–72.1)
3 years	50.7 (43.4–57.6)	80.0 (20.4–96.9)	54.1 (46.5–61.0)
4 years	44.7 (37.4–51.7)	80.0 (20.4–96.9)	48.3 (40.7–55.5)
5 years	40.8 (33.5–47.9)	80.0 (20.4–96.9)	44.8 (37.2–52.1)
Combined Surgery and Chemotherapy Survival			
	Overall	Age ≤18	Age >18
1 year	84.7 (78.1–89.5)	80.0 (20.4–96.9)	86.0 (79.5–90.6)
2 years	67.2 (59.1–74.1)	80.0 (20.4–96.9)	70.3 (62.3–76.9)
3 years	56.6 (48.2–64.1)	80.0 (20.4–96.9)	60.1 (51.6–67.5)
4 years	51.1 (42.7–59.0)	80.0 (20.4–96.9)	54.5 (45.8–62.3)
5 years	46.9 (38.4–54.9)	80.0 (20.4–96.9)	50.9 (42.2–58.9)

Table S2. Survival data of 4507 Patients with Mucoepidermoid Carcinoma (MEC) from the Surveillance Epidemiology and End Result (SEER) Database for Combination Therapy.(2000–2018).

**Combined Surgery, Radiation, and Chemotherapy Survival (%, 95% Confidence Interval)**		
With Radiation Prior to Surgery		
	Age ≤18	Age >18
1 year		60.0 (12.6–88.2)
2 years		60.0 (12.6–88.2)
3 years	*	60.0 (12.6–88.2)
4 years		60.0 (12.6–88.2)
5 years		40.0 (5.2–75.3)
With Radiation After Surgery		
	Age ≤ 18	Age > 18
1 year	100.0	89.8 (83.1–93.9)
2 years	100.0	75.2 (66.4–82.0)
3 years	100.0	66.0 (56.6–73.9)
4 years	100.0	60.0 (50.3–68.4)
5 years	100.0	57.8 (48.0–66.4)
With Radiation Before and After Surgery		
	Age ≤ 18	Age > 18
1 year		50.0 (0.6–91.0)
2years		50.0 (0.6–91.0)
3years	*	50.0 (0.6–91.0)
4 years		50.0 (0.6–91.0)
5 years		0.0

*No pediatric patients received a combination of all three therapies when radiation was given prior to surgery or when given before and after.

Table S3. Survival data of 4507 Patients with Mucoepidermoid Carcinoma (MEC) from the Surveillance Epidemiology and End Result (SEER) Database by Demographics (2000–2018).

Survival	Male %	Female %	White %	Black %	American Indian-Asian-Pacific Islander %
1 year	91.4 (89.9–92.6)	95.7 (94.6–96.6)	92.6 (91.5–93.6)	94.5 (91.8–96.4)	97.6 (95.4–98.7)
2 years	85.0 (83.2–86.7)	93.0 (91.6–94.1)	87.7 (86.3–88.9)	90.6 (87.2–93.1)	94.1 (91.0–96.1)
3 years	80.2 (78.1–82.1)	89.7 (88.1–91.1)	83.1 (81.5–84.6)	87.5 (83.7–90.4)	91.9 (88.4–94.3)
4 years	77.0 (74.8–79.0)	88.2 (86.4–89.7)	80.6 (78.9–82.1)	85.3 (81.2–88.5)	90.2 (86.5–92.9)
5 years	73.7 (71.4–75.9)	86.5 (84.6–88.2)	77.7 (75.9–79.4)	83.2 (78.9–86.7)	89.4 (85.6–92.3)
